# Evaluation of Male Infertility Prevalence with Clinical Outcomes in Middle Anatolian Region

**DOI:** 10.7759/cureus.5122

**Published:** 2019-07-10

**Authors:** Ünal Öztekin, Mehmet Caniklioğlu, Sercan Sarı, Volkan Selmi, Abdullah Gürel, Levent Işıkay

**Affiliations:** 1 Urology, Bozok University Faculty of Medicine, Yozgat, TUR

**Keywords:** male infertility, prevalence, primary infertility

## Abstract

Objective: the aim of this study is to determine the prevalence of male factor infertility with the clinical patterns of patients in our region.

Materials and methods: this is a descriptive retrospective study of 406 infertility cases presented at our urology clinic from February 2018 to February 2019. We assessed hormone and physical examination data, semen analysis results, the contribution of male and female factors to infertility, and types of infertility (i.e., primary or secondary).

Results: the age of the male patients ranged from 18 to 50 years, with a mean of 30 ± 5 years. Asthenozoospermia was the leading cause of male factor infertility in 77 patients (19%). Male factors as the sole cause of infertility were found in 185 (45.6%) couples. Female factors as the sole cause were found in 32 couples (7.9%). Primary infertility was determined in 314 (77.3%) patients, and 92 (22.7%) had secondary infertility.

Conclusion: according to our results, the male infertility rate was high among couples reporting infertility. Couples should be informed about the causes of infertility, which may be due factors attributed to either sex.

## Introduction

Infertility is the inability for a couple to achieve pregnancy after one year of regular and unprotected sexual intercourse. Infertility can cause significant financial loss and emotional stress affecting one in seven people, or roughly 49 to 72 million people worldwide. Infertility affects both men and women, and approximately 10% to 15% of couples in industrialized countries are infertile [1]. In approximately half of all cases, infertility is caused by male-related factors [2]. In 50% of childless couples, abnormal sperm parameters are the male infertility factor. However, in 30% to 40% of infertile couples, male infertility factors are absent. Infertility in the absence of abnormal sperm is idiopathic male infertility and may be caused by several factors such as endocrine failure, reactive oxygen species, and genetic abnormalities. Primary infertility is seen in one in eight couples; secondary infertility is seen in one in six couples [3]. Primary infertility was defined as failure to conceive after one year of unprotected sexual intercourse in a couple trying to achieve a pregnancy who had not previously conceived, and secondary infertility was defined infertility following a previous pregnancy. Clinical-based studies indicate that most infertile couples seek clinical help for primary infertility, whereas population-based studies indicate an equal or higher proportion of couples with secondary infertility [4].

Azoospermia, oligozoospermia, asthenozoospermia, teratozoospermia, and mixed pathology (oligoasthenoteratozoospermia) are abnormal sperm parameters that cause male infertility. Ovulatory disorders, tubal blockage, uterine abnormality, peritoneal factors, and endometriosis are the common causes of female infertility [5].

The aim of this study is to determine the prevalence of male factor infertility with the clinical patterns of patients in our region.

## Materials and methods

This is a descriptive retrospective study of patients who were admitted to our urology clinic for infertility from February 2018 to February 2019. Outcomes of patients were retrieved from medical records and infertility forms. After receiving approval from the local ethics committee, patients provided written informed consent and were evaluated retrospectively in accordance with the Declaration of Helsinki (2017-KAEK-189_2019.02.28_18). Records of 476 patients were obtained; however, 70 of the records were incomplete. So, 406 patients were included in the study. Demographic and clinic datas of the patients such as age, BMI, primary or secondary infertility status (couples with children were accepted as secondary infertility), smoking, presence of varicocele, testicular volumes, reproductive hormone levels, and sperm parameters were recorded. Testis and vascular structures were evaluated with color Doppler ultrasonography and recorded. These couples have had regular and unprotected sex for at least one year without achieving the desired pregnancy. General physical examinations were performed, and hormonal tests were conducted to measure total testosterone, luteinizing hormone (LH), follicle stimulating hormone (FSH), prolactin, and estradiol. Varicocele grades were recorded; Grade 1 was defined as the varicocele only be palpated during Valsalva maneuver, Grade 2 was easily palpable but not visible, and a Grade 3 varicocele was easily visible [6]. Patient semen analysis was conducted at the same laboratory after at least three days of sexual abstinence. Semen analysis was evaluated according to the World Health Organization 2010 criteria [7]. Patients were grouped according to their sperm analysis as normozoospermic, azoospermia, oligozoospermia, asthenozoospermia, mixed pathology (oligoasthenoteratozoospermia). Sociodemographic data, reproductive hormone levels, testis volumes (measured with a Prader orchidometer), previous infertility treatment history (e.g., antioxidant treatment, varicocelectomy, testicular sperm extraction, intrauterine insemination, and in vitro fertilization) were evaluated separately for each group. Descriptive analyses were performed and recorded.

## Results

The duration of infertility ranged from one to 23 years, with a median of 1.5 years. Forty-six male patients (11.3%) had a family history of infertility.

The age of the male patients ranged from 18 to 50 years, with a mean of 30 ± 5 years. The mean BMI at presentation was 26.6 ± 4.1. Two hundred patients were smokers (49.3%). Fifty couples (12.3%) had previous abortus. Five patients (1.2%) patients had Grade 1 varicocele, 177 (43.6%) had Grade 2, and 58 (14.3%) had Grade 3. Mean right and left side testis volumes were 16.4 ± 3.0 ml and 16.3 ± 3.3 ml, respectively. In the azoospermic group, the right and left side testis volumes were 12.2 ± 5.2 ml and 11.7 ± 5.6 ml, respectively. The mean FSH level in the azoospermic group was 14.6 ± 12.4 mUI/ml and 5.1 ± 5.9 mUI/ml in all patients. (Table 1).

**Table 1:** Demographic, hormone, and physical examination data Abbreviations: SD - standard deviation; BMI - body mass index; TT - total testosterone; LH - luteinizing hormone; FSH - follicle stimulating hormone; PRL - prolactin; E2 - estradiol; IUI - intrauterine insemination; IVF - in vitro fertilization; TESE - testicular sperm extraction.

	Azoospermia (n=42; 10.3%)	Oligozoospermia (n=22; 5.5%)	Asthenozoospermia (n=77; 19.0%)	Mixed pathology (n=68; 16.7%)	Normozoospermia (n=197; 48.5%)	All patients (N=406)
Age, mean±SD	31.64±6.92	29.50±4.10	30.34±6.22	31.78±6.01	30.36±5.17	30.6±5.6
BMI, mean±SD	27.4±3.617	26.37±4.66	26.34±4.13	26.77±4.66	26.67±3.92	26.6±4.1
Smoker Status						
Yes, n (%)	23 (54.8%)	11 (50.0%)	37 (48.1%)	34 (50.0%)	95 (48.2%)	200 (49.3%)
No, n (%)	19 (45.2%)	11 (50.0%)	40 (51.9%)	34 (50.0%)	102 (51.8%)	206 (50.7%)
Abortus history, n (%)	1 (2.4%)	2 (9.1%)	6 (7.8%)	8 (11.8%)	33 (16.8%)	50 (12.3%)
Varicocele, n (%)						
Absence	17 (40.5%)	10 (45.5%)	32 (41.6%)	21 (30.9%)	86 (43.7%)	166 (40.9%)
Grade 1	0 (0.0%)	0 (0.0%)	1 (1.3%)	0 (0.0%)	4 (2.0%)	5 (1.2%)
Grade 2	23 (54.8%)	10 (45.5%)	23 (29.9%)	30 (44.1%)	91 (46.2%)	177 (43.6%)
Grade 3	2 (4.8%)	2 (9.1%)	21 (27.3%)	17 (25.0%)	16 (8.1%)	58 (14.3%)
Testis volume, mean±SD						
Right (ml)	12.2±5.2	16.5±2.0	17.1±1.7	15.3±3.1	17.5±1.6	16.4±3.0
Left (ml)	11.7±5.6	16.0±2.6	17.2±1.7	15.2±3.7	17.3±1.7	16.3±3.3
Hormone level, mean±SD						
FSH (mUI/ml)	14.6±12.4	4.2±2.4	3.7±2.2	6.2±5.3	3.4±2.1	5.1±5.9
LH (mUI/ml)	6.5±4.5	3.8±2.2	3.5±1.5	4.4±3.0	3.3±1.5	3.9±2.5
PRL (µg/lt)	11.9±16.7	10.3±5.0	11.5±7.9	13.05±20.9	9.09±3.1	10.5±11.0
E2 (ng/lt)	24.7±22.2	20.0±8.3	24.8±9.7	25.3±11.4	25.8±26.2	25.1±20.7
TT (ng/dl)	356.4±185.9	442.4±158.5	477.1±194.8	509.3±214.7	471.1±162.5	465±184
Previous Infertility treatment, n (%)						
Antioxidant	0 (0.0)	1 (4.5)	6 (7.8)	6 (8.8)	7 (3.6)	20 (5)
Varicocelectomy	1 (2.4)	2 (9.1)	13 (16.9)	8 (11.8)	5 (2.5)	29 (7.1)
IUI	1 (2.4)	0 (0.0)	2 (2.6)	1 (1.5)	2 (1.0)	6 (1.4)
IVF	14 (33.3)	1 (4.5)	0 (0.0)	4 (5.9)	6 (3.0)	25 (6.1)
TESE+	4 (9.5)	0 (0.0)	0 (0.0)	0 (0.0)	1 (1.5)	5 (1.2)
TESE-	10 (23.8)	0 (0.0)	0 (0.0)	0 (0.0)	0 (0.0)	10 (2.4)

Table 2 shows the type of abnormality found in the male patients. Forty-two patients (10.3%) had azoospermia, and 22 (5.4%) had oligozoospermia. Asthenozoospermia was the leading cause of male infertility in 77 patients (19%). Mixed pathology (oligoasthenoteratozoospermia) was seen in 68 cases (16.7%). Semen analysis results were unremarkable in 197 cases (48.5%). The accountable causes of the azoospermia for our patients were ductus deference agenesia in two patients, hormonal disorders (e.g., hypogonadism and hyperprolactinemia) in 28 patients, orchitis in one patient and chemotherapy in one patient. Mean FSH and LH level in azoospermia group was higher than the others, and the mean testosterone level was low. Also, mean testicle sizes were smaller.

**Table 2:** Results of semen analysis of patients applying for infertility investigation

Sperm Abnormality	Frequency	Percentage
Azoospermia	42	10.3
Oligozoospermia	22	5.4
Asthenozoospermia	77	19.0
Mixed pathology	68	16.7
Normozoospermia	197	48.5

Table 3 shows the relative contribution of both male and female partners to the identifiable causes of infertility in 406 couples. Infertility due to male factors was found in 185 (45.6%) couples. Infertility due to female factors accounted for 32 cases (7.9%). Twenty-four couples (5.9%) had a combination of male and female factors. Unexplained infertility accounted for 165 cases (40.6%).

**Table 3:** Contribution of male and female factors to infertility

Etiological factor	Frequency	Percentage
Male only	185	45.6
Female only	32	7.9
Both partners	24	5.9
Unexplained	165	40.6

Of the 406 couples, 314 (77.3%) had primary infertility, and 92 (22.7%) had secondary infertility (Table 4).

**Table 4:** Types of infertility

Infertility Type	Frequency	Percentage
Primary	314	77.3
Secondary	92	22.7

## Discussion

Although the worldwide prevalence of infertility seems to be stable at approximately 9% to 12%, in Turkey, the infertility rate has declined significantly by 46%, from 15.0% in 1993 to 8.1% in 2013 [1]. Over the past 20 years, approximately 30% to 50% of cases of infertility are due to male factors, and 20% of cases are due to a combination of both male and female factors. Infertility due to female factors occurs in 50% to 70% of cases [8-10]. Male infertility due to semen quality has declined worldwide [11]. In our study, male factors accounted for infertility in 45.6% of couples, while female factors accounted for 7.9% of infertility cases. In our region, traditionally, women are held responsible for infertility, and couples usually seek clinical gynecologic aid first. The gynecologist then recommends couples seek the counsel of a urologist. Therefore, our female factor infertility rate was low.

The primary infertility prevalence was high in our region (77.3%), which aligns with similar findings in previous studies [12,13], but differed from studies conducted in other countries [5,14]. Eighty-one men (20%) received treatment due to infertility, and 37.0% of couples underwent intrauterine insemination or in vitro fertilization (IVF), indicating a strong desire to have children. Günay et al. evaluated 252 couples and reported the IVF rate of 11.1% [15]. Increased use of Assisted Reproductive Technology with governmental incentives adopted after 2005 may be the cause of the declining prevalence of infertility.

When we evaluated abnormal sperm parameters as the cause of male infertility, asthenozoospermia was the most common pathology (16.7%). Asthenozoospermia is a condition characterized by reduced sperm motility in semen and affects approximately 19% of infertile men [16]. Prolonged sexual abstinence, sperm dysfunction, varicocele, genital tract infections, genetic factors, and unhealthy lifestyle are the most common etiological factors that cause reduced sperm motility [17]. Azoospermia may be as high as 20% among male infertility cases, although it is seen in approximately 1% of male populations [18,19]. In our study, the azoospermia rate was 10.3% in all patients and 20.0% among the patients with abnormal sperm parameters. Karabulut et al. evaluated the patients admitted to three infertility clinics in Turkey, and the azoospermia rate was 5.85% among the 9,733 patients in that study (18.3% of patients with abnormal sperm parameters) [20].

Varicocele is associated with the increase in the temperature of testicles, and the prevalence of clinically relevant varicocele associated with infertility and decreased sperm quality is 5% to 20%. Arteriolar vasoconstriction causing testicular hypoxia may occur because of the high concentrations of adrenal cortical hormones in refluxing blood and cause damage in the seminiferous epithelium [21-23]. Male infertility due to varicocele was 42.7% [24]. One study determined that varicocele was the major cause of male infertility [22].

Some societies associate the number and gender of children with a woman’s status or success [15]. If a couple has children, it reflects positively on both the man and the woman. In infertile couples, the woman is frequently held responsible for the issues of infertility. Our study provides evidence to the contrary; in most of the couples diagnosed with primary infertility, there was a high prevalence of male-related infertility. Varicocele, orchitis, bilateral ductus deference agenesis, cancer treatment, and hormonal disorders were the main factors for male infertility. Asthenozoospermia was the most common cause of male infertility, and most Grade 3 varicocele cases were in this group.

This study has some inherent limitations due to its retrospective design. The data of the patients who applied to the obstetrics outpatient clinic were insufficient and genetic analysis of the patients were not available.

## Conclusions

Male factor infertility is a significant contributor to couple infertility in our region. Patients should be educated on infertility as a partially curable condition and understand its many causes, which includes factors from both sexes.
